# Connecting myelin-related and synaptic dysfunction in schizophrenia with SNP-rich gene expression hubs

**DOI:** 10.1038/srep45494

**Published:** 2017-04-06

**Authors:** Hedi Hegyi

**Affiliations:** 1 CEITEC - Central European Institute of Technology, Masaryk University, 62500 Brno, Czech Republic

## Abstract

Combining genome-wide mapping of SNP-rich regions in schizophrenics and gene expression data in all brain compartments across the human life span revealed that genes with promoters most frequently mutated in schizophrenia are expression hubs interacting with far more genes than the rest of the genome. We summed up the differentially methylated “expression neighbors” of genes that fall into one of 108 distinct schizophrenia-associated loci with high number of SNPs. Surprisingly, the number of expression neighbors of the genes in these loci were **35 times** higher for the positively correlating genes (**32 times** higher for the negatively correlating ones) than for the rest of the ~16000 genes. While the genes in the 108 loci have little known impact in schizophrenia, we identified many more known schizophrenia-related important genes with a high degree of connectedness (e.g. *MOBP, SYNGR1* and *DGCR6*), validating our approach. Both the most connected positive and negative hubs affected synapse-related genes the most, supporting the synaptic origin of schizophrenia. At least half of the top genes in both the correlating and anti-correlating categories are cancer-related, including oncogenes (*RRAS* and *ALDOA*), providing further insight into the observed inverse relationship between the two diseases.

Gene expression correlation, protein-protein interaction and other high-throughput experiments in the post-genomic era have revealed that genes tend to form complex, scale-free networks where most genes have a few connections with others and a few have a high number of interactions, commonly referred to as “hubs”, establishing them as important central genes in these gene networks^1^. These highly interconnected genes have become the targets of intense research expecting them to play prominent roles in genetic diseases. However, measurements found only a weak correlation between disease genes and hubs, e.g. Barabasi *et al*.^2^ found that disease genes have 32% more interactions with other proteins than non-disease genes, arguing that genetic mutations in topologically central, widely expressed genes are more likely to result in severe impairment of normal development, leading to lethality in utero and eventual deletion from the population^2^.

This is apparently not the case in schizophrenia, a disease that steadily affects about one percent of the population despite the lower fecundity of the affected individuals^3^. In most cases of schizophrenia no known mutations exist in protein-coding genes^4^ and the role of gene expression dysregulation in schizophrenia is increasingly recognized^5^,^6^.

A recent meta-analysis, examining the mutations in the genomes of 36,000 schizophrenics, identified 108 distinct genomic regions with significantly higher mutation rates^7^. We integrated this set with gene expression data from the Allen Brain Atlas^8^,^9^. After determining the correlating and anti-correlating neighbors of genes in the 108 loci we found that the median number of correlating neighbors were about **35 times** higher for the genes in the 108 regions than in the rest of the genome (**32 times** for the anti-correlating pairs).

We also integrated the data with a recent methylome study in schizophrenics^10^. Ranking the genes for the hypermethylated probes of the positively correlating gene pairs identified the top gene as *SYNGR1*, a synapse-related gene whose regulatory region overlaps with one of the 108 SNP-rich loci in ref. 7. Ranking the genes for the hypermethylated probes of the negatively correlating gene pairs identified *MBP* and *MOBP*. They are both myelin-related, anti-correlating with a large number of synapse-related and glutamate receptor genes, offering a model that connects these frequently observed but so far disjoint pathologies in schizophrenia.

## Results

### Genes in the most mutated genomic regions have the highest number of correlating partners

In the first step we identified all human gene expression pairs with a Pearson correlation of >=0.8 or <=−0.7 in human brain tissues using the Allen Brain Atlas (website: brain-map.org)^8^,^9^, and a recent database with pre-calculated Pearson correlations for all relevant gene pairs at http://www.szdb.org/download.html#coexpression by Wu *et al*.^11^. This resulted in 1,257,407 positively correlating and 1,108,585 negatively correlating unique gene pairs belonging to 16829 and 16761 individual genes, respectively (Supplementary Tables 1 and 2).

Subsequently we mapped all known human genes in 108 highly mutated human genomic regions identified in ref. 7 separately for genes whose promoters or only *cis* regulatory elements fall into these 108 loci. Filtering for genes present in the correlating or anti-correlating pairs by Wu *et al*.^11^ resulted in 254 promoter-selected and 462 cis-selected genes (indicated by 1p/1c in Supplementary Tables 1 and 2, respectively).

**Figure 1:**
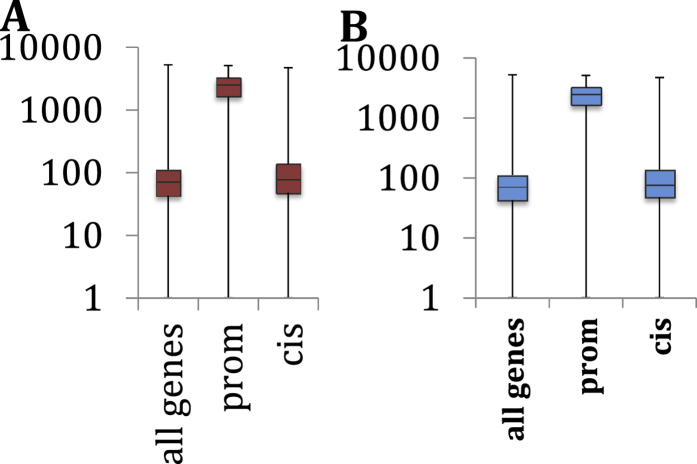
Boxplots of inteacting partners for all genes and promoter-selected and cis-selected genes in 108 SNP-rich genomic regions taken from ref. 7. (**A**) Positive, (**B**) negative correlating partners.

Counting the positively and negatively correlating partners for all the genes except in the 108 loci in ref. 7 resulted in a median number of 71 positively and 63 negatively correlating pairs, respectively (Fig. 1). Unexpectedly, the median numbers of correlating pairs for the promoter-selected genes were 2472 and 2013, corresponding to a **35**-fold and **32**-fold increase for the positive and negative pairs, respectively.

The number of correlating pairs for the *cis*-selected genes also proved to be higher (386 and 320 pairs on average for positively and negatively correlating pairs, respectively), corresponding to an approximately 2.5-fold increase when compared to the general gene population. The median values were similar to the average gene population in this category (Fig. 1) but an unpaired t-test showed that the *cis*-derived genes and the general gene population (excluding the 108-loci genes) are significantly different (p*-*value = 0.012).

We also investigated the number of differentially methylated gene pairs using a table of 56001 differentially methylated probes from a genome-wide methylome study in schizophrenics^10^. We counted separately the number of hypermethylated and hypomethylated genes for the highly correlating and anti-correlating gene pairs and also the total sum of hyper- and hypomethylated probes belonging to these genes. The sums of scores for both the number of differentially methylated gene pairs and the total sum of their differentially methylated probes are shown in Supplementary Tables 1 and 2, for the positively and negatively correlating pairs, respectively.

**Figure 2:**
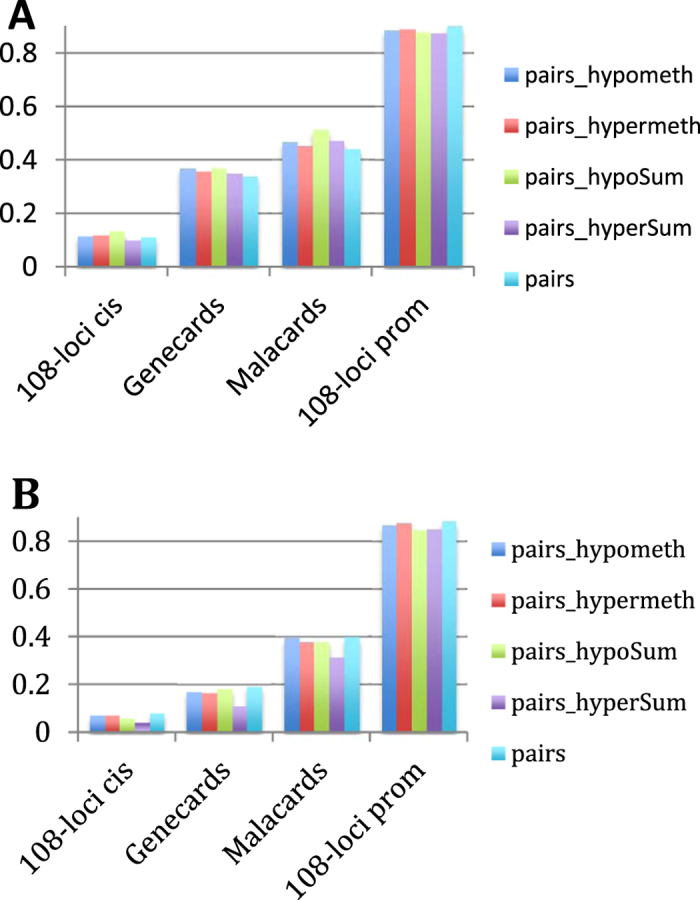
The effect sizes for the ratios of gene neighbor numbers between specific genes and their complementary gene sets (i.e. the rest of the ~16 k genes present in the study): 108-loci cis genes; Malacards-annotated schizophrenia genes; Genecards-annotated schizophrenia genes; 108-loci promoter genes. Effect sizes were calculated for 5 different measurements: (i) **pairs**, the total number of correlating gene pairs; (ii) **pairs_hypometh**, the number of correlating gene pairs that are hypomethylated (defined as in at least one probe the gene is hypomethylated); (iii) **pairs_hypermeth**, the number of correlating gene pairs that are hypermethylated; (iv) **pairs_hypoSum**, the total number of hypomethylated probes of the correlating gene pairs; (v) **pairs_hyperSum**, the total number of hypermethylated pairs for the correlating gene pairs. The numbers were filtered using only pairs of genes where both genes were differentially methylated in ref. 10. (**A**) Positively, (**B**) Negatively correlating gene pairs.

To determine which measurement distinguishes the best between schizophrenia-related and non-specific genes we calculated the effect size for the 108-loci genes and also for genes annotated by Genecards and Malacards^12^,^13^ as schizophrenia-related (Fig. 2), using Cliff’s delta for five different measurements (see legend for details).

Figure 2a and b shows the effect sizes for all relevant pairs of complementary gene sets (i.e. promoter-derived *vs.* non-promoter-derived, Genecards *vs.* non-Genecards genes, etc.) comparing the numbers of correlating gene partners in one set to the numbers of the gene partners in the other gene set. As expected the promoter-derived 108-loci genes had the greatest effect size, followed by Malacards and Genecards, the 108-loci cis-derived genes having the smallest effect. The results were similar for the negatively correlating pairs (Fig. 2b). Here we also filtered for gene pairs that were both differentially methylated in ref. 10, which increased the effect size by 0.039 for “pairs” in Fig. 2 for the promoter-derived positive set and by 0.061 for the same for the negative set. (Unfiltered charts shown in Supplementary Figure 1A,B).

### Specific genes with high numbers of correlating partners

We ranked all genes according to the number of hypermethylated probes (taken from ref. 10) of the correlating gene partners. Tables 1 and 2 show the top 20 genes with the most hypermethylated probes, in the positively and negatively correlating partners, respectively. The top-ranking gene in Table 1 is *SYNGR1*, synaptogyrin, followed by *GRIN1*, a glutamate receptor and *CHRNB2*, a cholinergic receptor. All three genes have synapse-related functions, *SYNGR1* and *GRIN1* regulate synaptic plasticity whereas *CHRNB2* regulates synapse assembly^14^.

*SYNGR1* is also present in the 108 most mutated loci in ref. 7 although only for its regulatory region, not for its promoter. Further functional analysis identified 5 more synapse-related genes in Table 1: *NISCH* regulates synaptic transmission, *RIMS1* and *STX1A* regulate postsynaptic potential, *CPLX2* regulates synaptic plasticity and *GRIA1* regulates long-term synaptic depression.

Altogether 11 out of the 20 genes in Table 1 are annotated in Genecards as schizophrenia-related and 14 appear in the 108 genomic regions in ref. 7. The only gene in Table 1 that appears in neither is *ARHGEF11*, a glutamate transport enhancer, however it had a significantly higher expression in the thalamus of schizophrenics in ref. 15.

Table 2 lists the top 20 genes with the highest number of hypermethylated probes for the negatively correlating partners. The top-ranking gene, *DBI*, is a GABA receptor modulator; its role having been contemplated in schizophrenia^16^ the authors concluded that *DBI* might have a symptom modulatory rather than an etiological role in schizophrenia. Unexpectedly, *MOBP*, myelin-associated oligodendrocyte basic protein and *MBP*, myelin basic protein, both appear in Table 2. While myelin-related abnormalities are one of the hallmarks of schizophrenia, surprisingly, not much functional information is available about *MOBP* beyond its role in the formation of the myelin sheath^17^ and a knockout mouse was phenotypically indistinguishable from the wild type^18^. Nevertheless, the authors argue that *MOBP* probably has a so far undiscovered function, due to the conservation of several alternatively spliced variants in rat and mouse^18^.

Altogether, 11 of the 20 genes with the greatest numbers of hypermethylated probes for their anti-correlating gene partners (Table 2) are annotated as schizophrenia-related in Genecards and 10 appear in the 108 loci in ref. 7.

Interestingly, several genes in both tables are cancer-related (indicated with a “*” next to the gene identifiers in Tables 1 and 2). Among the top 20 genes for hypermethylated probes of correlating partners are *ARHGEF11*^19^,^20^ and *SREPF2*, both associated with prostate and breast cancer^21^,^22^ while *ALDOA* functions as an oncogene in highly metastatic pancreatic cancer^23^. *NISCH* has a tumor-suppressive function in breast cancer^24^, *CHGB* is associated with aggressive VHL-associated pancreatic neuroendocrine tumors^25^. *SEZ6L2* is a prognostic marker for lung cancer^26^, *SLC45A1* is deleted in neuroblastoma^27^. *GRIA1* might play a role in glioma proliferation^28^ while *TCF20* is again associated with prostate^29^ and breast cancer^30^.

Among the top 20 genes for hypermethylated probes of anti-correlating partners are *RRAS*, an oncogene, also involved in neuronal axon guidance^31^ and *MBP*, associated with oligodendrogliomas^32^. *TMEM219* regulates apoptosis^33^; *CDK2AP1* is a putative oral cancer suppressor^34^; *PSMB10*, a proteasome subunit, is upregulated via the NFKB1 pathway in cancer cells^35^. *S100A1* is also associated with several tumor types and inhibits apoptosis in ventricular cardiomyocytes^36^.

*DGCR6* is associated with DiGeorge syndrome, a consequence of microdeletions in chromosomal region 22q11.2 and also has increased levels in metastatic mammary tumour cells^37^. *FABP7* plays a role in neurogenesis and is a marker of glioma stem cells^38^. Epithelial splicing regulatory protein 2 (*ESRP2*) suppresses cancer cell motility^39^. *ALDH1A1* is a marker and prognostic factor for several human cancers, bladder^40^, pancreatic^41^, lung^42^, colorectal and breast cancer^43^, among others.

Altogether, 10 genes among the top 20 for the greatest number of hypermethylated probes for the positively correlating gene partners (Table 1) and 13 of the 20 genes with the greatest numbers of hypermethylated probes for their anti-correlating gene partners (Table 2) are annotated as cancer-related in Genecards.

### The connection between the positive and negative hubs

**Figure 3:**
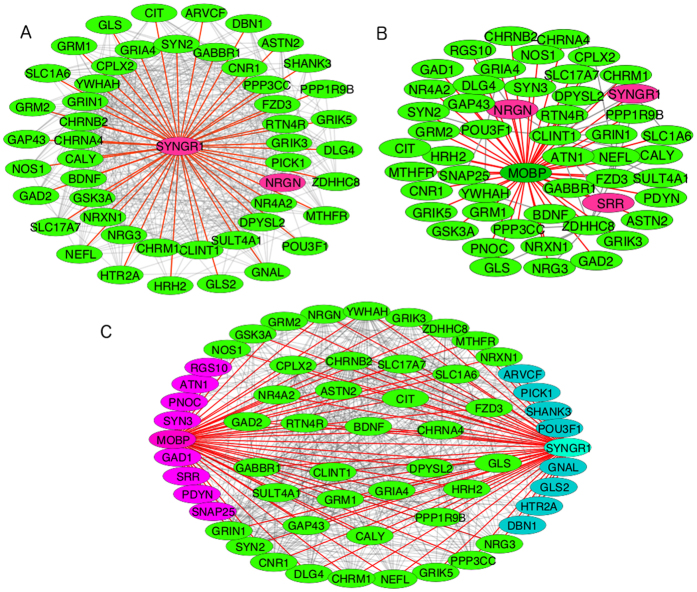
Gene networks of hub genes generated from hypermethylated Malacards-annotated genes. (**A**) SYNGR1-centered, positively correlating gene network. The red-colored edges show correlations with SYNGR1. (**B**) Negatively correlating gene network, centered on MOBP. All the red-colored edges show negative correlations with MOBP. All the other pairwise negative correlations are shown in grey. The purple-colored genes overlap with 108 highly mutated genomic loci in schizophrenics (see text for details). (**C**) The combined network in (**A** and **B**). All light green-colored genes correlate positively with SYNGR1 and negatively with MOBP. The purple-colored and turquoise-colored genes correlate only with MOBP or SYNGR1, respectively.

The existence of the two kinds of hub genes with high numbers of positive or negative correlating partners raises the question about their functionality and the underlying neurobiological pathways: are they related or do they form mostly separate networks? To answer this question we constructed three gene networks (Fig. 3): two representing the positive and negative correlations only for *SYNGR1* and *MOBP*, respectively (Fig. 3a,b), and one (Fig. 3c) showing a combined network of the two. We chose *SYNGR1* for the positive hub for it is the top ranking gene in Table 1 and is also located in one of the PGC regions whereas we chose MOBP because it ranks highly among the anti-correlating genes, indicating a putative regulatory function, which, however, has not been observed yet experimentally.

To reduce the size of the networks we selected only Malacards-annotated schizophrenia-related genes that are hypermethylated in ref. 10. *SYNGR1* correlates positively with 49 genes (Fig. 3a); *MOBP* also has 49 negatively correlating partners (Fig. 3b). The combined network is shown in Fig. 3c. Strikingly, 41 genes are shared interacting partners between *MOBP* and *SYNGR1*. Each of the two hub genes interacts with only 8 genes that are not shared with the other hub. Clearly, the positive and negative correlations - and such interactions – form highly interconnected networks that provide synaptic functions, the core functionality of the human brain.

The shared genes reflect remarkably on the nature and most consistently observed features of schizophrenia: 6 shared genes are glutamate receptors (*GRIA4, GRIK3, GRIK5, GRIN1, GRM1, GRM2*), one GABA receptor (*GABBR1*), a cannabinoid receptor (*CNR1*), *GAD2*, a glutamate decarboxylase, two synapse-related (*SYN2, SYT11*) and several neuron-specific genes are also present among the shared genes (Fig. 3c).

**Figure 4:**
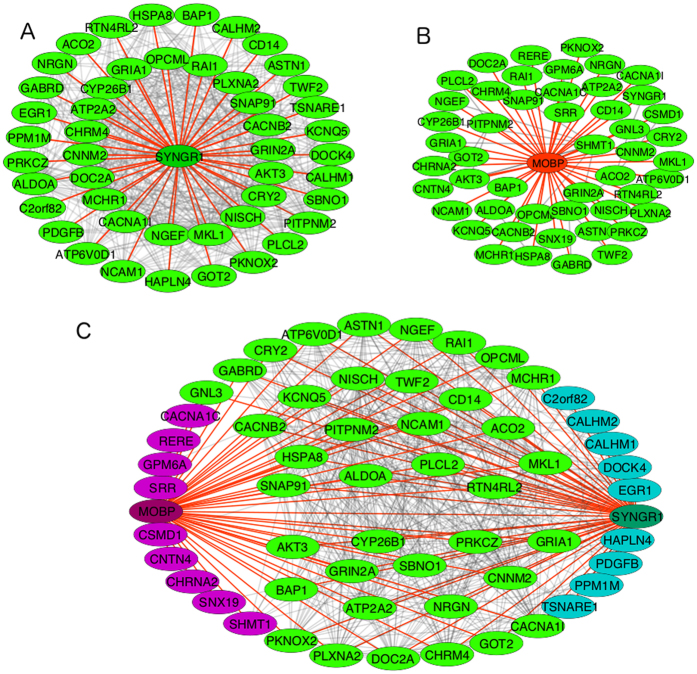
(**A**) All positively correlating genes in the 108 loci. The network is again centered on *SYNGR1*. The red-colored edges show correlations with *SYNGR1*. (**B**) All the genes in the 108 loci that correlate negatively with *MOBP* (edges colored red) or one another (grey edges). (**C**) The combined network in (**A** and **B**). All light green-colored genes correlate positively with *SYNGR1* and negatively with *MOBP* (edges show in red in both cases). The purple-colored and turquoise-colored genes correlate only with *MOBP* or *SYNGR1* (of the two), respectively.

We repeated the selection process replacing the Malacards-genes with the 108-loci genes. This resulted in a similar number of interacting genes that correlate positively with *SYNGR1* (50 genes) and negatively with *MOBP* (48 genes). They share 39 common genes (Fig. 4). Biological processes derived from a Gene Ontology^44^ analysis of the shared genes either in the Malacards set or the 108-loci set and in both cases shared between *SYNGR1* and *MOBP* and significantly enriched (p-value < 0.05) for both sets are shown in Table 3. For both gene sets the top two biological processes with the highest significance are “synaptic transmission” and “modulation of synaptic transmission”.

### Functional analysis of schizophrenia genes correlating with the top 20 hub genes

To get a further insight into the biological processes the top ranking genes and their network neighbors (i.e. their correlating and anti-correlating gene partners) partake in we took the top 20 genes with the most hypermethylated probes for the correlating (and anti-correlating) gene partners and selected those gene neighbors that correlated with *minimum* 19 of the top 20 genes. This resulted in 421 genes for the positively correlating set and 460 genes for the negatively correlating one.

For this *in silico* functional analysis we used an online tool provided by STRING^45^ to identify Gene Ontology terms that are over-represented in our gene set. The positive gene set was associated with 278 significantly enriched GO terms for biological processes while the negative set had 151 such GO terms. Interestingly, while the two gene sets share only 117 common genes i.e. about 1/4^th^ of the total gene number for either set, they also share 117 common biological processes (Table 4), a much higher fraction for either set (42% of the positive set-derived biological processes and 77% of the negative set-derived processes are shared).

This result also shows that gene networks as a whole are remarkably redundant, i.e. similar functions are often carried out by several functional homologs in the brain. This network view of the genes associated with schizophrenia is also revelatory for the polygenic nature of schizophrenia: once a mutation perturbs the expression of a highly connected hub gene with many interacting partners, this in turn will lead to the perturbation of several hundred or even thousand interacting genes. The differential methylation of several thousands of genes in the prefrontal cortex of schizophrenics might be one such manifestation of the complexity of the disease.

The shared GO terms ranked by significance are listed in Table 4. The top process is “nervous system development”, followed by “synaptic transmission”, reflecting two major aspects of schizophrenia, i.e. it is considered a (neuro)developmental disease^46^ affecting mostly the synapse^47^. On a different note, the most frequently occurring word in Table 4 is “regulation” (occurring 29 times), followed by “transport” (24x), perhaps underlining somewhat less pronounced aspects of schizophrenia.

### Positive hub genes are longer, negative hub genes are shorter than average

We also calculated the protein length and protein disorder statistics for both the top 20 positive and negative hubs. Surprisingly, while the top 20 positive hub proteins were significantly longer (p-value < 10^−5^) than the average human protein (894 and 442 amino acids, on average, respectively), the top 20 negative hub proteins were significantly shorter (327 amino acids on average). We did not find their disorder to be significantly different from that of the general human protein population. Likewise, the 108 region-derived proteins among the top 20 positive and negative hubs in Tables 1 and 2, were also longer (14 proteins in Table 1a with an average length of 983 amino acids) and shorter (10 proteins in Table 1b with an average length of 261), respectively, than the average human protein (442 aa).

## Discussion

It has been known for at least a decade that myelin has an inhibitory role in axonal regeneration^48^ in the CNS. Myelin is dysfunctional in schizophrenia^49^ and this dysfunctionality leads to changes in synaptic formation and function, another hallmark of schizophrenia^49^. Several studies have identified genes whose expression is abnormal in schizophrenic brains affecting myelin-related^50^ and synapse-related biochemical pathways^47^. However, this is the first time, to our knowledge, that a model for a complete network of gene interactions is presented that would account for both of these recurring anomalies in schizophrenia.

Our gene network has two hubs, one, *SYNGR1*, with a synaptic function, correlates positively and apparently interacts with a high number of genes that also interact with one another and at the same time interact negatively with *MOBP*, a myelin gene. Myelin is known to inhibit axonal sprouting, a step considered important for synaptic formation^51^. While neither *MOBP*, nor *MBP* are known to have such inhibitory functions, another myelin-related protein, *RTN4* (also called Nogo-A), ranking 88th among the negatively correlating genes (Supplementary Table 2), does have such a function, inhibiting axon growth^51^. It is tempting to hypothesize that either *MOBP* or *MBP* also have such a - so far undiscovered - inhibitory function. *MOBP* is the 3^rd^ most abundant protein in the CNS myelin and has several alternatively spliced variants. As highlighted by Montague *et al*.^18^ a physiological function for *MOBP* has not been found yet. Our model with two antagonistic hub genes would also account for the antagonistic relationship between myelin genes and synapse-related functions^52^.

We also found a high representation of cancer-related genes in both the top correlating and anti-correlating genes (Tables 1 and 2). While only limited association has been found so far connecting schizophrenia- and cancer-related gene hubs^53^, there are several studies pinpointing the essential role of hub proteins in cancer^2^,^54^. Only a handful of papers have been published on gene networks in schizophrenia, e.g. a study highlighting shared hubs and gene networks between schizophrenia and diabetes^55^ and our own work on microRNA-driven networks and hub genes in schizophrenia^56^. Perhaps the most relevant study connecting cancer- and schizophrenia-related gene networks is by Ibanez *et al*.^57^ who found an inverse comorbidity between the two diseases, observing expression deregulations in opposite directions of the same genes and gene networks.

Our model is also supported by the extraordinary enrichment of correlating partners for the genes encoded in the 108 genomic regions with the highest mutation rates in the genomes of schizophrenics identified in ref. 7. As mentioned above, the median number of correlating partners for those genes whose promoters are located in the 108 loci is 35 times higher for the positively correlating genes than for the rest of the genes in this study and 32 times higher for the negatively correlating genes. The effect size for these comparisons is in the range of 0.8–0.9, making the number of interacting partners, reflecting the centrality of a gene, the single most important factor when considering the biological significance of the individual schizophrenia-related genes and their contribution to the disease. There is a significant but smaller enrichment (2.5 times on average) for those 108-loci genes whose promoters are not, only their enhancers are present in these loci, which raises the intriguing possibility that for most genes in this set the mutations fall into or close to the promoter regions, compromising their functionality as in most cases of schizophrenia the protein sequences are not corrupted by mutations.

The assumption that it is the regulatory regions, not the protein-coding regions that are affected mostly in schizophrenia is also apparent in the fact that despite the strong centrality (“hubness”) of the affected genes we do find surviving phenotypes, which are the patients, exactly. As raised in the introduction, hub genes with a mutation in the coding region would make these mutations lethal in most instances^2^.

The robustness of our findings is also supported by the fact that when we replace the Malacards genes with the 108-loci genes in Fig. 3 (sharing only *SYNGR1, SRR* and *NRGN* as indicated in Fig. 3) and analyze the Gene Ontology terms for the 39 genes correlated positively by *SYNGR1* and negatively by *MOBP*, we find that the most significant biological process associated with this gene set is again “synaptic transmission”. This also shows the remarkable redundancy of the gene networks in the human brain. Altogether, beyond providing an intriguing new model for schizophrenia, with more details for the underlining gene networks than before, it is also fascinating and quite fitting that synaptic transmission, perhaps the most complex and dynamic part of the human brain also entails the most genetic complexity, i.e. the most connected gene networks with the highest number of correlating/interacting gene partners.

## Methods

### Determining pairwise gene expression correlations in the human brain

In the first step all pairwise Pearson correlations were determined for those genes expressed in the brain that have expression data in the Allen Brain Atlas (website: brain-map.org)^8^,^9^. Gene expression was measured for 50,000 genes in 524 different tissues taken from several compartments of the brains of several individuals spanning the human lifetime between 2 weeks of post-conception and 40 years of age. We used an in-house Perl script to calculate pairwise correlations complemented by correlation data taken from SZDB.org^11^. We filtered the results keeping only pairwise Pearson correlation that were either minimum 0.8 or maximum −0.7. If there were several values for the same gene pair we used the most extreme ones, the highest and lowest values for the positive and negative correlations, respectively.

We mapped all known human genes in 108 highly mutated human genomic regions identified in ref. 7 separately for genes whose promoters or only *cis* regulatory elements fall into these 108 loci. We determined the location of the promoters and cis elements in the human genome using a study by Thurman *et al*.^58^.

### Counting correlating gene pairs and their differentially methylated probes

For each gene whose expression correlated (r >= 0.8) or anti-correlated (r <= −0.7) with other genes in our data set (16830 and 16762 genes with correlating and anti-correlating partners, respectively) we counted the number of correlating and anti-correlating partners. Using a methylome data set in ref. 10 that recorded 56001 differentially methylated probes between two subgroups of schizophrenia patients we also counted gene partners for each gene that were hypermethylated or hypomethylated (with at least one hypermethylated or hypomethylated probe, respectively) and also the total number of hyper- or hypomethylated probes for the correlating and anti-correlating gene partners. The genes were ranked for the total sum of hypermethylated probes of the ‘neighboring’ (i.e. either correlating or anti-correlating) genes as this ranking placed *SYNGR1*, a gene present with its regulatory region in the 108 SNP rich regions in schizophrenics, identified by the Psychiatric Genomics Consortium (PGC) to the top of the list (in Table 1). This ranking also produced 14 genes out of the top 20 genes in Table 1 with either its promoter or cis-regulatory region overlapping with the SNP-rich PGC regions in ref. 7.

### Determining the effect size to distinguish between schizophrenia-related and unrelated genes

To determine which measurement distinguishes the best between schizophrenia-related and non-specific genes we calculated the effect size for the 108-loci genes and also for genes annotated by Genecards and Malacards^12^,^13^ as schizophrenia-related (Fig. 2) regarding each specific feature mentioned in the previous paragraph (i.e. the number of correlating genes, the number of hypermethylated, hypomethylated correlating genes and the total sum of the differentially methylated probes of the correlating pairs). We repeated the calculations for the negatively correlating pairs as well. To calculate the effect size we used Cliff’s delta. Cliff’s delta is defined as


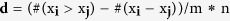


where the two distributions are of size **m** and **n** with items **x**_**i**_ and **x**_**j**_ (with ***i*** running from **1 to m** and ***j*** running from **1 to n**) respectively, and # is defined as the number of times. Cliff’s delta shows that out of all possible pairwise comparisons between the numbers in set **A** and set **B** in what proportion will the numbers in set **A** be bigger than in set **B**. Cliff’s delta does not require any assumptions about distribution types.

### Functional analysis with the Gene Ontology module of STRING

To carry out functional analysis of the top 20 genes and their network neighbors we used the Gene Ontology (GO) module^14^ of the STRING^45^ webserver. The server lists all functional categories that are significantly enriched in the provided gene set, and supplies the corresponding p-values. We recorded the biological processes for the top 20 positive and negative genes and also their network neighbors.

### Network visualization, statistical calculations

To visualize the gene correlation networks in Figs 3 and 4 Cytoscape^59^ was used. To carry out t-test calculations and calculate the corresponding p-values we used cpan’s Statistics package, a Perl library. Whenever not mentioned explicitly, calculations and data manipulation was carried out with in-house Perl script (available on request from the author).

## Additional Information

**How to cite this article**: Hegyi, H. Connecting myelin-related and synaptic dysfunction in schizophrenia with SNP-rich gene expression hubs. *Sci. Rep.* **7**, 45494; doi: 10.1038/srep45494 (2017).

**Publisher's note:** Springer Nature remains neutral with regard to jurisdictional claims in published maps and institutional affiliations.

## Supplementary Material

Supplementary Information

Supplementary Table 1

Supplementary Table 2

## Figures and Tables

**Table 1:** The top 20 positively correlating genes, ranked according to the hypermethylated probes (listed in column 6) in the correlating gene partners.

gene	pairs	pairs hypo meth	pairs hyper meth	pairs hypo Sum	pairs hyper Sum	probes hypo	probes hyper	genecards Score	malacards Score	prom/ cis?	prot len	description
**SYNGR1**	4336	1437	2086	3553	8290	0	8	**8.57**	NA	**1c**	233	synaptogyrin 1
**GRIN1**	3800	1261	1915	3302	7959	2	1	**9.07**	7.55	NA	959	glutamate receptor, ionotropic, NMDA 1
**CHRNB2**	4070	1391	1992	3465	7880	1	3	**9.07**	5.10	NA	502	cholinergic receptor, nicotinic beta 2
ARHGEF11*	4024	1394	1951	3506	7832	0	3	NA	NA	NA	1562	Rho guanine nucleotide exchange factor 11
ALDOA*	4949	1558	2208	3983	7811	2	5	**−2.01**	NA	**1p**	418	aldolase, fructose-bisphosphate A
SREBF2*	3750	1295	1810	3352	7643	1	0	**−1.79**	NA	**1c**	1141	sterol regulatory element binding TF 2
**PITPNM2**	3308	1202	1734	3188	7626	2	18	**−2.01**	NA	**1p**	1349	phosphatidylinositol transfer protein
GRAMD1B	4159	1428	1980	3418	7569	2	6	NA	NA	**1c**	745	GRAM domain containing 1B
NISCH*	4152	1331	1951	3250	7495	0	3	**−1.60**	NA	**1c**	1504	nischarin
RIMS1*	4679	1474	2067	3461	7465	0	3	NA	NA	**1c**	1692	regulating synaptic membrane exocytosis 1
**CPLX2**	3433	1149	1677	3139	7345	3	5	**9.07**	2.48	NA	134	complexin 2
CHGB*	3665	1205	1746	3154	7316	1	0	**9.07**	3.83	NA	677	chromogranin B
**STX1A**	3941	1259	1880	3023	7283	1	6	**−0.60**	NA	NA	288	syntaxin 1 A
SEZ6L2*	3406	1169	1747	3046	7265	0	1	NA	NA	**1c**	923	seizure related 6 homolog (mouse)-like 2
L3MBTL2	4406	1361	1972	3127	7227	1	0	NA	NA	**1p**	705	l(3)mbt-like 2 (Drosophila)
SLC45A1*	3454	1160	1740	2988	7218	1	4	NA	NA	**1c**	782	solute carrier family 45 member 1
GRIA1*	4337	1377	1950	3256	7141	0	3	**9.06**	NA	**1c**	916	glutamate receptor, ionotropic, AMPA 1
RANGAP1	3704	1232	1772	3074	7091	0	1	NA	NA	**1p**	587	Ran GTPase activating protein 1
TCF20*	3497	1242	1700	3258	7044	0	1	NA	NA	**1p**	1960	transcription factor 20 (AR1)
EPC2	5124	1514	2084	3436	6909	1	0	NA	NA	**1p**	807	enhancer of polycomb homolog 2

The total number of positively correlating genes (column 2), of which hypo- or hypermethylated (columns 3 and 4), are also listed. Genecards and Malacards scores are listed in columns 7 and 8. The column “prom/cis?” indicates if the promoter of the gene (“1p”) or the cis regulatory region of the gene (“1c”) falls into the 108 highly mutated loci in ref. 7. A “*” next to the gene identifier indicates a role in cancer.

**Table 2:** The top 20 negatively correlating genes, ranked according to the hypermethylated probes (listed in column 6) in the anti-correlating gene partners.

gene	pairs	pairs hypo meth	pairs hyper meth	pairs hypo Sum	pairs hyper Sum	probes hypo	probes hyper	genecards Score	malacards Score	prom/ cis?	protlen	description
DBI*	6013	1882	2601	4400	9376	NA	NA	**−1.01**	NA	NA	143	diazepam binding inhibitor (GABA receptor modulator)
C1orf54	5656	1792	2507	4233	9164	0	1	NA	NA	1p	131	chromosome 1 open reading frame 54
RRAS*	6426	1851	2669	4197	8986	1	0	NA	NA	NA	218	related RAS viral (r-ras) oncogene homolog
**C2orf82**	6837	1948	2720	4274	8934	2	1	**−0.01**	NA	1p	121	chromosome 2 open reading frame 82
NDUFA4L2	6517	1837	2635	4132	8668	5	1	NA	NA	1p	93	NADH dehydrogenase 1 alpha subcomplex, 4-like 2
**MOBP**	5741	1678	2462	3622	8576	9	0	**9.07**	2.40	NA	206	myelin-associated oligodendrocyte basic protein
MBP*	5467	1656	2349	3644	8266	25	8	**8.56**	NA	NA	304	myelin basic protein
CDK2AP1*	4638	1496	2120	3668	8130	5	0	**−0.79**	NA	1p	115	cyclin-dependent kinase 2 associated protein 1
S100A1*	4791	1529	2114	3501	7497	1	0	NA	NA	NA	147	S100 calcium binding protein A1
FABP7*	3921	1299	1852	3224	7490	3	1	**0.00**	NA	NA	166	fatty acid binding protein 7, brain
TMEM219*	5188	1603	2153	3795	7218	NA	NA	NA	NA	1p	240	transmembrane protein 219
DGCR6*	5993	1721	2310	3836	7040	0	1	**10.48**	13.8	NA	220	DiGeorge syndrome critical region gene 6
ESRP2*	3767	1188	1770	2842	6828	0	1	NA	NA	1p	727	epithelial splicing regulatory protein 2
**AS3MT**	3711	1269	1718	3120	6780	0	2	**0.22**	NA	1p	375	arsenite methyltransferase
ANP32E	3907	1204	1747	3092	6738	1	0	NA	NA	1p	268	acidic nuclear phosphoprotein 32 family member E
ALDH1A1*	3992	1236	1815	2898	6720	NA	NA	**−1.00**	NA	NA	501	aldehyde dehydrogenase 1 family member A1
PSMB10*	5214	1565	2058	3579	6528	NA	NA	NA	NA	1p	273	proteasome subunit beta 10
HIRIP3	5503	1514	2183	3158	6524	NA	NA	NA	NA	1p	556	HIRA interacting protein 3
SELENBP1*	5044	1426	2090	2952	6514	4	0	**0.15**	NA	NA	514	selenium binding protein 1
RENBP*	5201	1438	2111	2956	6507	NA	NA	**−0.60**	NA	NA	427	renin binding protein

The total number of positively correlating genes (column 2), of which hypo- or hypermethylated (columns 3 and 4), are also listed. Genecards and Malacards scores are listed in columns 7 and 8. The column “prom/cis?” indicates if the promoter of the gene (“1p”) or the cis regulatory region of the gene (“1c”) falls into the 108 highly mutated loci in ref. 7. A “*” next to the gene identifier indicates a role in cancer.

**Table 3:** Biological processes shared between the Malacards gene set interacting with both *SYNGR1* and *MOBP* in Fig. 3C (41 light green-colored genes) and the 108-loci genes interacting with both *SYNGR1* and *MOBP* in Fig. 4C (39 light green-colored genes).

GO_identifier	GO_Biological Process	shared Genes_108 loci	p_val (108 loci)	shared Genes Malacards	p_val (Malac Genes)
GO:0050804	modulation of synaptic transmission	7	0.00359	14	1.23e-13
GO:0007268	synaptic transmission	9	0.00359	16	8.05e-12
GO:0061564	axon development	7	0.0318	11	2.14e-06
GO:0031175	neuron projection development	8	0.0289	11	1.08e-05
GO:0048167	regulation of synaptic plasticity	4	0.0469	6	4.71e-05
GO:0048666	neuron development	9	0.0233	11	4.71e-05
GO:0048812	neuron projection morphogenesis	7	0.0318	9	0.000146
GO:0007411	axon guidance	6	0.0415	7	0.00128
GO:0030182	neuron differentiation	9	0.0318	9	0.00545
GO:0044765	single-organism transport	15	0.0318	15	0.0062
GO:0007399	nervous system development	14	0.0138	12	0.011
GO:1902578	single-organism localization	16	0.0289	15	0.0111

12 biological processes in the table are common in the two shared sets, despite the very limited number of actually shared genes (only *NRGN* is shared besides *MOBP* and *SYNGR1* but these two were not included in the GO analysis).

**Table 4:** Biological processes shared between the positively correlating and negatively correlating gene neighbors of the top 20 genes in Table 1 and Table 2.

GO_identifier	GO_Biological Process	Pos Neighbors	p_val (Pos Neigh)	Neg Neighbors	p_val (Neg Neigh)
GO:0007399	nervous system development	88	5.21e-12	105	4.23e-17
GO:0007268	synaptic transmission	48	2.95e-14	49	7.45e-14
GO:0048699	generation of neurons	68	1.57e-11	70	3.36e-10
GO:0048666	neuron development	52	6.61e-12	52	3.78e-10
GO:0031175	neuron projection development	48	2.83e-12	46	9.22e-10
GO:0022008	neurogenesis	68	1.62e-10	70	2.03e-09
GO:0007267	cell-cell signaling	57	2.83e-12	53	1.32e-08
GO:0048731	system development	99	0.000594	125	2.02e-08
GO:0030182	neuron differentiation	56	5.4e-11	54	2.31e-08
GO:0048667	cell morphogenesis involved in neuron differentiation	39	3.77e-10	38	2.98e-08
GO:0048812	neuron projection morphogenesis	39	7.5e-10	38	5.45e-08
GO:0061564	axon development	37	1.74e-09	36	1.16e-07
GO:0007409	axonogenesis	36	2.41e-09	35	1.49e-07
GO:0007275	multicellular organismal development	106	0.00347	132	7.65e-07
GO:0030030	cell projection organization	51	2.12e-08	50	1.66e-06
GO:0007610	behavior	32	1.34e-06	34	1.8e-06
GO:0048858	cell projection morphogenesis	42	1.1e-07	41	5.72e-06
GO:0007156	homophilic cell adhesion via plasma membrane adhesion molecules	10	0.0277	17	7.86e-06
GO:0048856	anatomical structure development	105	0.00619	128	1.01e-05
GO:0050803	regulation of synapse structure or activity	28	2.83e-12	20	1.43e-05
GO:0044767	single-organism developmental process	118	0.00225	138	2.61e-05
GO:0098742	cell-cell adhesion via plasma-membrane adhesion molecules	11	0.0369	18	2.65e-05
GO:0050804	modulation of synaptic transmission	27	2.94e-10	21	3.04e-05
GO:0044708	single-organism behavior	26	1.07e-05	27	3.14e-05
GO:1902580	single-organism cellular localization	37	0.000174	41	8.75e-05
GO:0007626	locomotory behavior	19	3.27e-06	18	0.000103
GO:0032502	developmental process	116	0.00628	136	0.000108
GO:0030001	metal ion transport	36	3.9e-07	33	0.00011
GO:0051650	establishment of vesicle localization	16	7.43e-05	17	0.000111
GO:0051640	organelle localization	25	5.23e-06	24	0.000152
GO:1902578	single-organism localization	116	2.83e-12	98	0.000153
GO:0006811	ion transport	46	8.77e-05	49	0.000157
GO:0007611	learning or memory	16	0.000443	18	0.000177
GO:0000902	cell morphogenesis	48	1.22e-07	43	0.000182
GO:0030534	adult behavior	15	3.73e-05	15	0.000195
GO:0044765	single-organism transport	110	7.01e-12	93	0.000195
GO:0050890	cognition	17	0.000535	19	0.000241
GO:0007417	central nervous system development	35	0.0013	40	0.000252
GO:0098660	inorganic ion transmembrane transport	34	1.76e-06	31	0.000405
GO:0051641	cellular localization	76	1.56e-06	73	0.000436
GO:0031344	regulation of cell projection organization	28	7.75e-06	26	0.000577
GO:0051656	establishment of organelle localization	22	6.8e-06	20	0.000577
GO:0061024	membrane organization	37	0.000377	39	0.000832
GO:0051960	regulation of nervous system development	38	6.8e-07	33	0.000962
GO:0048468	cell development	65	5.66e-07	59	0.00104
GO:0032989	cellular component morphogenesis	49	5.34e-07	43	0.00118
GO:0007420	brain development	28	0.00376	32	0.0012
GO:0006810	transport	125	2.83e-12	102	0.00145
GO:0043087	regulation of GTPase activity	29	6.67e-05	28	0.00148
GO:0010975	regulation of neuron projection development	24	6.8e-06	21	0.00152
GO:0045664	regulation of neuron differentiation	28	2.54e-05	26	0.00152
GO:0007613	memory	8	0.032	11	0.00167
GO:0051234	establishment of localization	126	6.68e-12	104	0.0017
GO:0030154	cell differentiation	89	0.00066	94	0.00201
GO:0048869	cellular developmental process	91	0.00124	97	0.00259
GO:0006836	neurotransmitter transport	11	0.00842	13	0.00268
GO:0044700	single organism signaling	139	1.56e-06	134	0.00275
GO:0051179	localization	146	2.83e-12	120	0.00276
GO:0071702	organic substance transport	66	3.73e-05	64	0.00276
GO:0060322	head development	28	0.00956	32	0.00351
GO:0065008	regulation of biological quality	94	1.07e-05	90	0.004
GO:1902582	single-organism intracellular transport	41	0.00307	44	0.00444
GO:0043547	positive regulation of GTPase activity	27	9.52e-05	25	0.00452
GO:0007154	cell communication	143	6.53e-07	135	0.00462
GO:0007411	axon guidance	25	3.26e-05	22	0.00462
GO:0034220	ion transmembrane transport	41	3.01e-06	35	0.00483
GO:0050767	regulation of neurogenesis	31	4.27e-05	28	0.00483
GO:0007269	neurotransmitter secretion	9	0.0209	11	0.00491
GO:0070838	divalent metal ion transport	16	0.00343	17	0.00515
GO:0023061	signal release	14	0.00137	14	0.00516
GO:0043269	regulation of ion transport	39	7.33e-09	28	0.00569
GO:0051649	establishment of localization in cell	68	9.18e-07	59	0.00675
GO:0006812	cation transport	35	0.000182	33	0.00683
GO:0035637	multicellular organismal signaling	10	0.00233	10	0.00764
GO:0050806	positive regulation of synaptic transmission	13	1.78e-05	10	0.00826
GO:0044093	positive regulation of molecular function	55	0.00211	57	0.00829
GO:0051336	regulation of hydrolase activity	51	2.23e-05	46	0.00829
GO:0048167	regulation of synaptic plasticity	17	1.64e-07	11	0.00978
GO:0098662	inorganic cation transmembrane transport	30	4.56e-06	24	0.0106
GO:0008088	axon cargo transport	9	5.65e-06	6	0.0132
GO:0072384	organelle transport along microtubule	7	0.000645	6	0.0132
GO:0008104	protein localization	62	2.35e-05	56	0.0139
GO:0045665	negative regulation of neuron differentiation	10	0.041	12	0.0139
GO:0007264	small GTPase mediated signal transduction	33	7.44e-05	29	0.0144
GO:0023051	regulation of signaling	76	0.00514	80	0.0146
GO:0031345	negative regulation of cell projection organization	10	0.00473	10	0.0146
GO:0001764	neuron migration	11	0.00124	10	0.0154
GO:0015031	protein transport	51	1.07e-05	44	0.0154
GO:0043085	positive regulation of catalytic activity	49	0.00174	49	0.0159
GO:0098655	cation transmembrane transport	33	1.16e-05	27	0.0159
GO:0010646	regulation of cell communication	88	5.43e-05	83	0.0163
GO:0010977	negative regulation of neuron projection development	8	0.0226	9	0.0163
GO:0045184	establishment of protein localization	53	1.32e-05	46	0.0166
GO:0001508	action potential	9	0.00602	9	0.0172
GO:0006816	calcium ion transport	14	0.00545	14	0.019
GO:0044802	single-organism membrane organization	28	0.00842	29	0.0227
GO:0033036	macromolecule localization	69	5.82e-05	63	0.0246
GO:0055085	transmembrane transport	49	1.53e-06	39	0.0251
GO:0035556	intracellular signal transduction	65	3.26e-05	58	0.0265
GO:0046907	intracellular transport	47	0.00101	45	0.0265
GO:0051899	membrane depolarization	7	0.01	7	0.0265
GO:0065009	regulation of molecular function	80	0.00064	78	0.027
GO:0007612	learning	10	0.01	10	0.0298
GO:0048489	synaptic vesicle transport	10	0.00296	9	0.0324
GO:0050773	regulation of dendrite development	11	0.00066	9	0.0324
GO:0032940	secretion by cell	22	0.00835	22	0.0356
GO:0051345	positive regulation of hydrolase activity	41	3.94e-06	32	0.0358
GO:0060284	regulation of cell development	36	5.56e-05	30	0.0365
GO:0097479	synaptic vesicle localization	11	0.000782	9	0.0365
GO:0050790	regulation of catalytic activity	67	0.00203	66	0.0383
GO:0050770	regulation of axonogenesis	10	0.014	10	0.0405
GO:0034765	regulation of ion transmembrane transport	31	1.05e-08	19	0.0418
GO:0050807	regulation of synapse organization	14	6.24e-06	9	0.0426
GO:0035725	sodium ion transmembrane transport	12	0.00121	10	0.0438
GO:0016043	cellular component organization	125	0.000226	121	0.0456
GO:0047496	vesicle transport along microtubule	4	0.0215	4	0.0457
GO:0051128	regulation of cellular component organization	76	3.59e-06	64	0.0495
