# Supplementary material for: Connecting myelin-related and synaptic dysfunction in schizophrenia with SNP-rich gene expression hubs
Source: Sci Rep. 2017 Apr 6;7:45494. doi: 10.1038/srep45494 (PMC5382542; doi:10.1038/srep45494)
Supplement: Supplementary Information [file srep45494-s1.doc]

Supplementary Information to “Connecting myelin-related and synaptic dysfunction in schizophrenia with SNP-rich gene expression hubs”

Hedi Hegyi, PhD*

CEITEC - Central European Institute of Technology, Masaryk University, 62500 Brno, Czech Republic

Tel: +420 549 494 278; Fax: +420 54949 2556;

Keywords: schizophrenia, methylation, synapse, myelination, gene networks.

*correspondence: hegyihedi@gmail.com

### Supplementary Information to “Connecting myelin-related and synaptic dysfunction in schizophrenia with SNP-rich gene expression hubs”

**Supplementary Figure 1**. The **effect sizes** for the ratios of gene neighbor numbers between **specific** genes and their **complementary** gene sets (i.e. the rest of the ~16k genes present in the study): 108-loci cis genes; Malacards-annotated schizophrenia genes; Genecards-annotated schizophrenia genes; 108-loci promoter genes. Effect sizes were calculated for 5 different measurements: (i) **pairs**, the total number of correlating gene pairs; (ii) **pairs_hypometh**, the number of correlating gene pairs that are hypomethylated (defined as in at least one probe the gene is hypomethylated); (iii) **pairs_hypermeth**, the number of correlating gene pairs that are hypermethylated; (iv) **pairs_hypoSum**, the total number of hypomethylated probes of the correlating gene pairs; (v) **pairs_hyperSum**, the total number of hypermethylated pairs for the correlating gene pairs. The measurements are the same as in **Figure 2** but here they are derived from all coexpression pairs in http://www.szdb.org/download.html#coexpression, not filtered for pairs of genes that are both differentially methylated in Ref. [1]. (**A**) Positively, (**B**) Negatively correlating gene pairs.


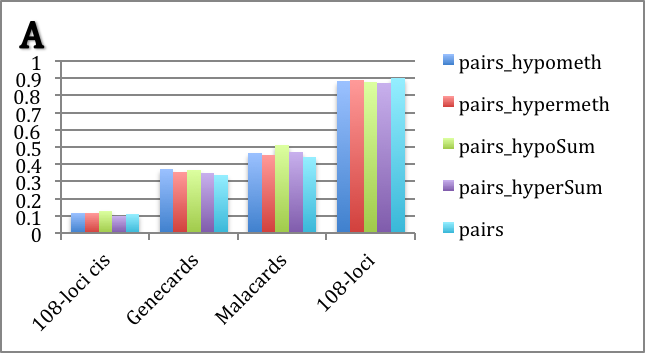

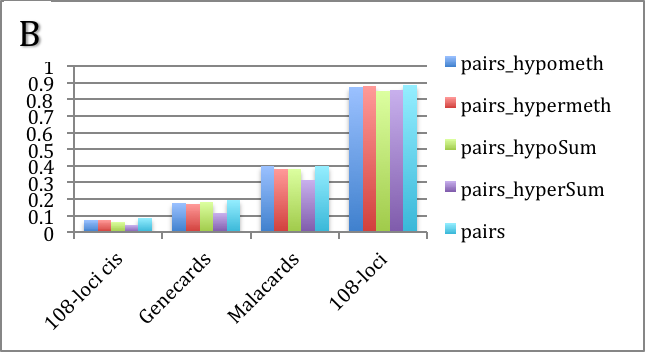


**Supplementary Table 1**. Summary information for all genes with positively correlating genes based on expression data in the Allen Brain Atlas [2, 3], pairwise gene expression correlations taken from [4], values downloaded from http://www.szdb.org/download.html#coexpression.

**Supplementary Table 2.** Summary information for all genes with negatively correlating genes based on expression data in the Allen Brain Atlas [2, 3], pairwise gene expression correlations taken from [4], values downloaded from http://www.szdb.org/download.html#coexpression.

## References

1. Wockner LF, Noble EP, Lawford BR, Young RM, Morris CP, Whitehall VL, Voisey J: **Genome-wide DNA methylation analysis of human brain tissue from schizophrenia patients**. *Transl Psychiatry* 2014, **4**:e339.

2. Hawrylycz MJ, Lein ES, Guillozet-Bongaarts AL, Shen EH, Ng L, Miller JA, van de Lagemaat LN, Smith KA, Ebbert A, Riley ZL *et al*: **An anatomically comprehensive atlas of the adult human brain transcriptome**. *Nature* 2012, **489**(7416):391-399.

3. Miller JA, Ding SL, Sunkin SM, Smith KA, Ng L, Szafer A, Ebbert A, Riley ZL, Royall JJ, Aiona K *et al*: **Transcriptional landscape of the prenatal human brain**. *Nature* 2014, **508**(7495):199-206.

4. Wu Y, Yao YG, Luo XJ: **SZDB: A Database for Schizophrenia Genetic Research**. *Schizophr Bull* 2016.
